# Method for Keyhole-Free High-Aspect-Ratio Trench Refill by LPCVD

**DOI:** 10.3390/mi13111908

**Published:** 2022-11-04

**Authors:** Henk-Willem Veltkamp, Yves L. Janssens, Meint J. de Boer, Yiyuan Zhao, Remco J. Wiegerink, Niels R. Tas, Joost C. Lötters

**Affiliations:** 1 MESA^+^ Institute for Nanotechnology, University of Twente, P.O. Box 217, 7500 AE Enschede, The Netherlands; 2 Bronkhorst High-Tech bv, Nijverheidsstraat 1A, 7261 AK Ruurlo, The Netherlands

**Keywords:** high-aspect-ratio trenches, keyhole-free refilling by LPCVD, trench isolation/insulation, 81.65.Cf, 81.15.-z, 81.15.Gh, 52.77.-j

## Abstract

In micro-machined micro-electromechanical systems (MEMS), refilled high-aspect-ratio trench structures are used for different applications. However, these trenches often show keyholes, which have an impact on the performance of the devices. In this paper, explanations are given on keyhole formation, and a method is presented for etching positively-tapered high-aspect ratio trenches with an optimised trench entrance to prevent keyhole formation. The trench etch is performed by a two-step Bosch-based process, in which the cycle time, platen power, and process pressure during the etch step of the Bosch cycle are studied to adjust the dimensions of the scallops and their location in the trench sidewall, which control the taper of the trench sidewall. It is demonstrated that the amount of chemical flux, being adjusted by the cycle time of the etch step in the Bosch cycle, relates the scallop height to the sidewall profile angle. The required positive tapering of 88° to 89° for a keyhole-free structure after a trench refill by low-pressure chemical vapour deposition is achieved by lowering the time of the etch step.

## 1. Introduction

In bulk MEMS [1,2,3,4,5], high-aspect-ratio (HAR) trench structures are used for electrical insulation [2,6,7], thermal insulation [8,9,10,11], the isolation during the fabrication of large cavities in the bulk of the silicon substrate [11], integration of silicon sidewall heaters in the trench-assisted surface channel technology (TASCT) [11], and the closure of buried channels in buried channel technology [12]. The sequence of HAR trench etching, the deposition of layers inside the trench, and the properties of the used materials determine the isolation/insulation properties and mechanical properties of the refilled trench. For the fabrication of HAR trench isolation/insulation structures, positively tapered trench sidewalls with sidewall profile angles of 88° to 89° [13] are required for a keyhole-free refill using low-pressure chemical vapour deposition (LPCVD) of materials such as silicon nitride (Si_*x*_N_*y*_), silicon dioxide (SiO_2_), and polycrystalline silicon (poly-Si), without a significant increase in surface topology, i.e., grooving at the wafer surface.

**Figure 1:**
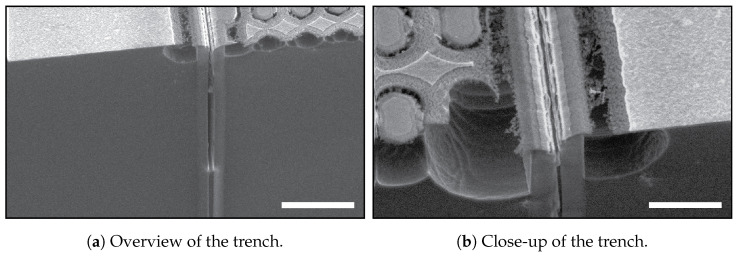
SEM images showing a failed channel etch in the TASCT process [11]. In (**a**), an overview, and in (**b**), a close-up of unwanted etching of the silicon surface in an SF_6_-based RIE step caused by a non-uniform photo-resist layer in the neighbourhood of a trench structure with an opened keyhole. The scale bar in (**a**) is 10 μm and in (**b**) is 5 μm.

Positively-tapered sidewalls in the trenches prevent the formation of the so-called keyholes, i.e., voids between the interfaces of conformally deposited layers at opposing sides of the trench generated by local width variations in that trench. The shape of the keyhole is often present over the complete length of the trench, and the width is in the order of 150 nm to 300 nm [11,14,15]. The presence of keyholes significantly reduces the mechanical strength, stability, and reliability of the MEMS structures [2,16], and complicates subsequent processing. Complications in the processing occur when, for example, the LPCVD layer on the wafer surface has to be stripped and the keyholes open up. The opened keyholes can then be accessed and affect subsequent steps, such as photolithography, (thin-)film deposition, and etching [11]. In Figure 1a,b, it is shown that an opened keyhole is responsible for a non-uniform photo-resist mask layer, resulting in destructive effects during a reactive ion etch (RIE) step with SF_6_ in the TASCT process. This non-uniformity is caused by the fact that photo-resist is pulled into the keyholes by capillary forces.

**Figure 2:**
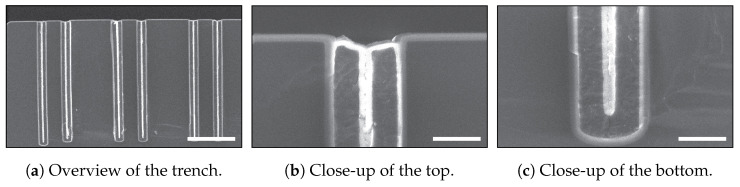
SEM images of the TASCT trenches after keyhole closure. This was achieved by etching the poly-Si at the wafer’s surface and closing the keyholes by dry thermal oxidation of poly-Si at 1100 ${}^{\circ }C$. In (**a**), the full trench is shown, in (**b**), a close-up at the top of the trench is shown, and in (**c**), a close-up at the bottom is shown [11]. The scale bar in (**a**) is 20 μm, and in (**b**,**c**) it is 2 μm.

In the work of Veltkamp et al., the fabrication of sidewall heating elements for fluidic applications by utilising keyhole-free refilled trenches fabricated in the TASCT process is presented [11]. In this work, the trenches were coated by means of thermal oxidation and refilled with LPCVD of poly-Si. The keyholes were eliminated by stripping the poly-Si layer on the wafer’s surface to open the keyhole and performing dry thermal oxidation of the poly-Si layer to completely fill the keyhole, as is shown in the overview image in Figure 2a and close-ups in Figure 2b,c. The uniformity of the closure was determined by the profile of the etched trenches and the diffusion of oxygen in the already formed t-SiO_2_ [17].

HAR trench structures in MEMS applications typically have widths in the range of 1 μm to 500 μm and are etched deep into the silicon or silicon-on-insulator (SOI) substrates using a deep reactive ion etching (DRIE) method. The aspect ratio of the etched trenches is often up to 30 [18]. The maximum width of a refilled trench is determined by the characteristics of the refill process (e.g., maximum layer thickness) and the properties of the deposited materials (e.g., internal stress). These demands together determine that the sidewall profile angle should be in the order of 88° to 89° [13]. The advantages of using DRIE of silicon are the flexibility in device design, the profile control along the depth of the trench, and the small footprint of the isolation/insulation structures that could be obtained. Bosch-based processes (also known as gas-chopping processes or time-multiplexed processes) are frequently used for DRIE of HAR isolation/insulation trenches into a silicon substrate [2,14,19,20,21].

**Figure 3:**
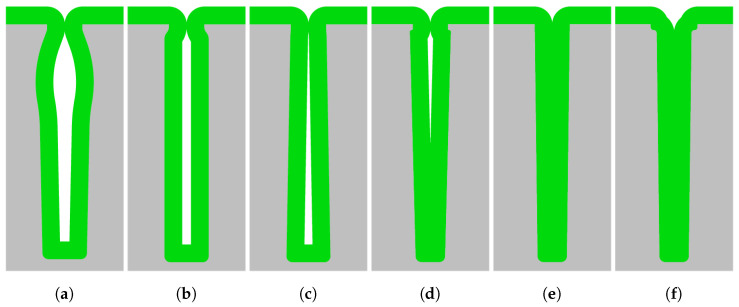
A schematic overview of refilled trenches with and without the trench entrance effect and various sidewall irregularities. In (**a**), a combination of the entrance effect with the bottling effect; in (**b**), a combination of the entrance effect with a 90${}^{\circ }$ directional profile in the trench; in (**c**), a negative tapered (re-entrant) profile; in (**d**), a combination of the entrance effect with a positively tapered profile; in (**e**), an ideally positively tapered profile without any entrance effect; and in (**f**), a positively tapered profile with increased widening at the top for an optimal conformal refill. Here, (**a**–**d**) result in keyhole formation after refill. In this image, the colours correspond to the following materials: ■ silicon and ■ refill material, such as Si_*x*_N_*y*_.

In Figure 3a–d, the most important DRIE profile irregularities, which are responsible for the formation of keyholes, are shown. The presence of keyholes is a combination of a local widening in the trench structure a few microns beneath the hard mask, the so-called entrance effect, and irregularities in the sidewall deeper into the etched trench. This entrance effect is a combined result of the hard mask etch and the chosen sequence and parameters of recipe steps in the trench etch. The sidewall irregularities can be classified as a local widening (bottling effect; see Figure 3a), a combination of the entrance effect with a uniform sidewall under-etch (Figure 3b), negatively tapered (re-entrant) sidewalls (Figure 3c), and a combination of the entrance effect and positively tapered sidewalls (Figure 3d).

In Figure 3e,f two successful iterations of refilling of the trenches as a result of positively-tapered sidewalls are shown. Figure 3e shows the ideal etch, and for Figure 3f, an entrance-effect-compensated pre-HAR etch step or sequence is used.

**Figure 4:**
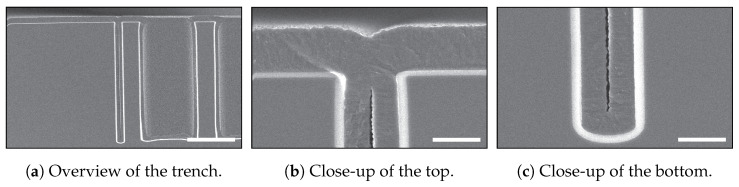
SEM images of refilled trenches in the TASCT process, in which a keyhole demonstrated the entrance effect. A 3 μm wide HAR trench was refilled by wet thermal oxidation and LPCVD of poly-Si. In (**a**), it is shown that the keyhole was created over the full length of the trench. In the close-up SEM images of (**b**,**c**), it is shown that the width of the keyhole was the same at the top and the bottom [11]. Dry thermal oxidation can be used to close these keyholes, as is demonstrated in Figure 2. The scale bar in (**a**) is 20 μm and in (**b**,**c**) is 2 μm.

In the work of De Jong et al. [14], various Bosch-based recipes were characterised with respect to profile determining features and their applicability in keyhole-free refilling. It was demonstrated that by using a Bosch-based recipe with one set of fixed parameters for etching the HAR trenches that it is difficult to eliminate the entrance effect and the irregularities (bottling effect) deeper inside the trench. Additionally, Veltkamp et al. [11] demonstrated that the entrance effect is often responsible for keyhole formation after a conformal refill of HAR trenches. It was shown (see Figure 4a) that despite having a perfect DRIE trench profile with 90${}^{\circ }$ sidewalls, a keyhole still formed over the complete length of the trench depth. In the close-up images in Figure 4b,c, it is shown that the width of the keyhole was the same at the top and the bottom. This demonstrates that the local entrance effect is responsible for the formation of the keyhole. Based on the work of De Jong et al. [14] and Veltkamp et al. [11], it can be concluded that it is very difficult to avoid the entrance effect and to control the sidewall profile for HAR trenches using one recipe with a fixed set of parameters for the trench etch.

In the work of Zhu et al. [2], it was demonstrated that by using a sacrificial layer of poly-Si, deposited on the wafer’s surface using LPCVD, the entrance effect can be avoided. The entrance effect is then localised in the poly-Si layer, which is stripped before the refill. They demonstrated also that the required directional or positive profile can be achieved by changing the ratio between deposition cycle time and etch-cycle time in the two-step Bosch-based process recipe. However, the described approach to eliminating the entrance effect uses additional processing steps, as poly-Si has to be deposited and patterned, which complicates the fabrication process.

In the work of Dixit et al. [22], Li et al. [23], and Yu et al. [20], a semi-isotropic SF_6_ post-etch was introduced to enlarge the entrance of the trench at the top of the surface. After the post-etch, the entrance of the trench is enlarged in the order of several microns, and therefore requires a thicker layer for refilling the trench. A three-step DRIE etch process has been developed by Nagarajan et al. for the tapering of silicon vias [24,25]. This method uses a Bosch-based DRIE step for etching the HAR trenches, a post-etch approach consisting of an RIE step to adjust the positive taper, and a final wafer-scale semi-isotropic etch step to smoothen the sidewalls. Major drawbacks of the post-etch method are that different plasma chemistries are used in the same plasma chamber to control the width and profile of the trench. The post-etch method is extremely sensitive to the remaining fluorocarbons (FC) inside the trench, originating from the used Bosch-based process. This may affect the etch uniformity in the trench and over the wafer and requires substantial overfilling for a perfect closure of the trench, or will make a successful refill not possible, as the maximum layer thickness is determined by the used refill equipment and materials. The etch rate of the post SF_6_-based etch is also sensitive to the aspect ratio of the trench and the exposed silicon area (i.e., loading effect), and therefore needs to be optimised for every mask layout.

Bosch-based processes are less sensitive to mask loading, making them robust processes which are nowadays often factory installed on DRIE etchers and frequently used in fields such as MEMS fabrication. Additionally, the processes are reliable, as they are less sensitive to wafer temperature variations. Those DRIE etchers are capable of running multi-step and/or multi-recipe Bosch-based processes with high performance (e.g., etch rate, sidewall profile, and mask selectivity) to etch HAR trenches and many other structures. This is realised by measuring in real-time the hardware performance of the inductively coupled plasma (ICP) and capacitively coupled plasma (CCP) generators, mass-flow controllers (MFCs), and automatic pressure control system. The real-time data are used to synchronise the hardware equipment during the FC deposition step, the directional removal of FC, and the semi-isotropic etching of silicon to guarantee reproducible etch performance. In the work of Chang et al. [26,27], it was demonstrated that a correctly tuned three-step Bosch-based process is capable of etching complex micro- and nano-structures by controlling the dimensions of the scallop size by using the so-called deposit, remove, etch, multistep (DREM) process.

**Figure 5:**
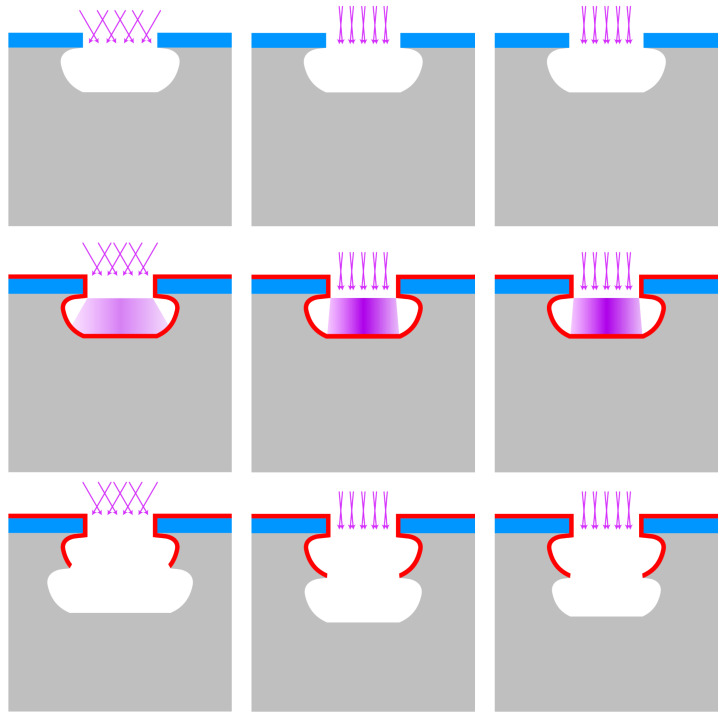

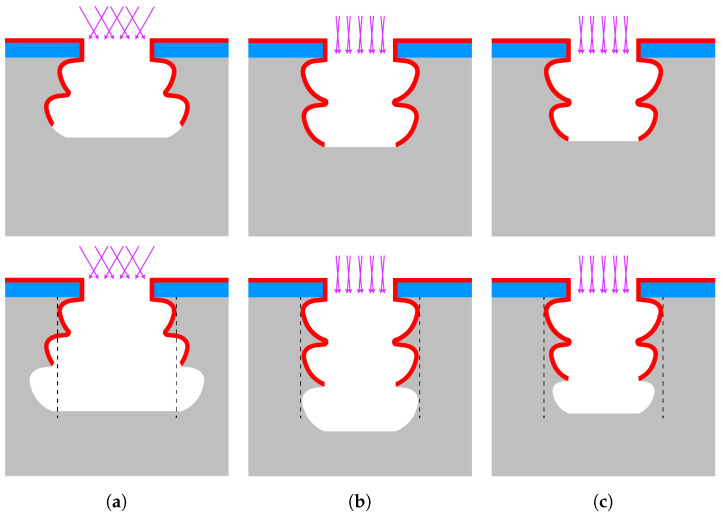
HAR profile control by scallop dimension and position tailoring by utilising the etch-cycle time and pressure (IAD). In (**a**), a negative trench profile is created by using a large IAD (etch pressure >30 mTorr) and constant etch-cycle time. In (**b**), a directional trench profile is created by using a small IAD (etch pressure <10 mTorr) and constant etch-cycle time [28,29]. In (**c**), a positive trench profile is created by using relatively short and constant (or ramped down) etch-cycle time and a small IAD. In this image, the colours correspond to the following materials: ■ silicon, ■ t-SiO_2_ hard mask, and ■ FC passivation layer. The purple colour in the second row indicates the IAD in the plasma. The concentration of ions is the highest in the centre (near perpendicular to the surface).

**Figure 6:**
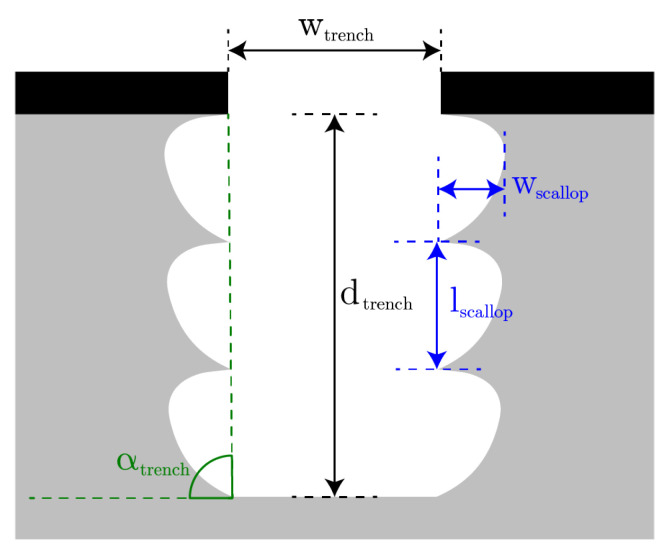
Schematics of an etched trench with a one-to-two ratio in lateral to vertical etching. In this work, the depth and width of the trench, the length and width of the scallop, and the angle of the trench were measured. These are denoted with $d_{trench}$, $w_{trench}$, $l_{scallop}$, $w_{scallop}$, and $\alpha_{trench}$, respectively.

In this paper, a study is presented of etching positively tapered HAR trenches by controlling the dimensions (i.e., height and width) of the scallops and their locations in the sidewall of the trench, while keeping grooving on the wafer’s surface to a minimum. The dimensions and locations of the scallops in the sidewall of the trench were adjusted by the removal of the FC layer at the bottom of the previous scallop and the etch cycle step of the new scallop, as is illustrated in the model in Figure 5 and which is explained in Section 2.2. In the removal of the FC layer at the bottom of the scallop, the ion angular distribution (IAD) and the ion energy determine the location and efficiency of the FC strip. In the etch cycle step, silicon is etched in a semi-isotropic manner with a two-to-one ratio between the vertical and lateral etch. The lateral etch, i.e., the width of the scallop ($w_{scallop}$, see Figure 6), is mainly created by the chemical flux (isotropic etching), and the vertical etch, i.e., the length of the scallop ($l_{scallop}$, see Figure 6), is a synergy of both the chemical flux (isotropic etching) and physical ion flux (directional etching). The ratio of those fluxes is controlled by the SF_6_ flow, the ICP power, the platen power, and the process pressure. The duration of those fluxes is controlled by the step time of the steps in the Bosch cycle. In this study, it is proposed that by tuning the dimensions of the scallops, the directionality of the profile angle of the trench sidewall can be tailored. The scallop’s dimensions were studied by varying the etch-cycle time, the etching process pressure, and the platen power during the etching step; and the resulting scallops were examined to achieve optimal DRIE-tailored sidewalls with profile angles in the range of 88° to 89° for trench refill. The scallop dimensions in the upper part of the trench sidewalls were used during characterisation. The reasons for this are twofold. Firstly, keyholes often form due to these scallops, as is shown by Veltkamp et al. [11]. Secondly, these scallops are not influenced by the mass transport of the species into the trench, and therefore contain the performance of etching process to that location inside the trench. Each of the varied parameters, the chosen settings, and their expected influences on the dimensions of the scallops etched in the HAR DRIE are discussed separately.

## 2. Influencing the Entrance Effect and Trench Profile

### 2.1. Trench Entrance Etch

**Figure 7:**
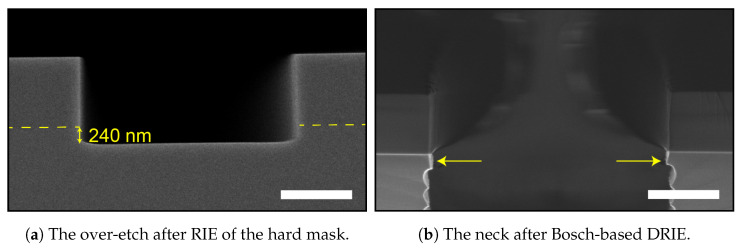
SEM images of the entrance effect. In (**a**) is the 240 nm deep over-etch into the silicon substrate during the RIE step to pattern the t-SiO_2_ hard mask layer. The dashed yellow line indicates the silicon–silicon-dioxide interface. In (**b**), the so-called “neck” is still visible after a Bosch-based DRIE and is indicated by yellow arrows. The scale bars in (**a**,**b**) are 1 μm.

A two-step Bosch-based process consisting of deposition and etch steps [30] normally starts with the deposition of FC. This FC layer acts as a protective layer for the mask material and prevents the formation of a large scallop/undercut directly beneath the mask [31]. A drawback of starting with a deposition step is creating a little silicon “neck” directly beneath the mask, as is shown in the scanning electron microscopy (SEM) image in Figure 7a. The origin of this neck or trench entrance effect in two-step Bosch-based recipes is in the removal of the FC layer using a physical flux of the F^+^ and SF_5_^+^ ions in the SF_6_ plasma, which are also etching the silicon [32]. This directional etch is achieved in the recipe by applying an increased platen power during the etch cycle, e.g., for 30% of the etch-cycle time.

In cases when a t-SiO_2_ hard mask is used, the formation of the neck can be enhanced by over-etching this t-SiO_2_ hard mask layer (Figure 7b) using a directional CHF_3_/O_2_-based RIE method. During this etch, the sidewall of the over-etched silicon is coated with FC and protects the silicon during the first scallop etch if no stripping is performed before the HAR DRIE.

**Figure 8:**
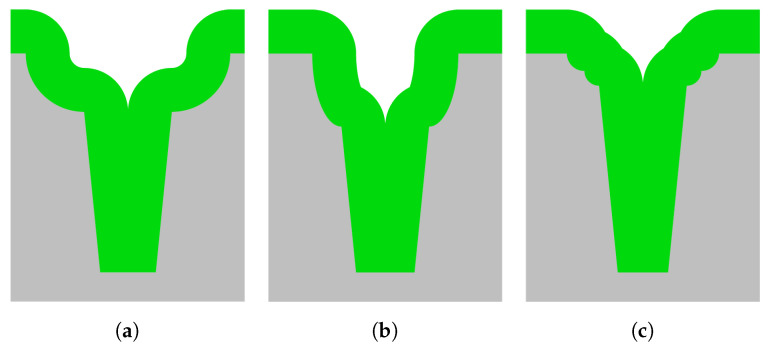
Schematic representations of refilled positively-tapered trenches with different entrances. (**a**) A high-pressure isotropic etch was used, which results in a rather wide grooving at the wafer surface after refilling. (**b**) A low-pressure semi-isotropic etch using a physical flux-assisted directional etch is used to etch the entrance, resulting in a rather deep grooving at the wafer surface. (**c**) A multi-step process using two high-pressure isotropic etch steps is used to etch the entrance. (**c**) The resulting grooving at the wafer surface is narrower and less deep. In this image, the colours correspond to the following materials: ■ silicon and ■ refill material, such as Si_*x*_N_*y*_.

The width and length of the first scallops in the opening of the trench are controlled by using a sequence of etch cycles based on the Bosch-based process. In Figure 8, three kinds of entrance etch profiles and their influences on a refill are illustrated. For Figure 8a,b, the entrances were etched using a single step based on an isotropic or semi-isotropic etch, respectively, resulting in either a wide or a deep entrance profile. Significant grooving at the wafer surface is obtained after refilling these trenches. Both types of grooving are non-ideal, as they form reservoirs, which will affect the quality of pattern transfer in subsequent photolithography, for example. The entrance shape in Figure 8c shows a multi-step approach consisting of two semi-isotropic etch steps, resulting in less grooving after refilling. Therefore, the demands of the required entrance profile for a perfect closure of the trench without significant grooving at the surface of the wafer can be defined as (1) the trench entrance needing to have a positive tapering, (2) the width not being meant to extend more than 1 μm outside the trenches, and (3) the depth not being meant to be more than 1 μm.

### 2.2. Trench Etch

To explain how the dimensions of the scallops and the trench profile angles are effected by the etch parameters, a short explanation is given for all three varied process parameters.

#### 2.2.1. Process Pressure and Ion Angular Distribution

The trench profile angle is mainly influenced by the ion impact of positively charged ions at the bottom of the trench. The ion impact determines the removal rate and efficiency of FC and the location where the FC is stripped (see Figure 5). In a plasma, the sheath electric field accelerates ions along the macroscopic surface, creating a directed flux of energetic particles which induces directional etching. Ion scattering in the sheath produces a distribution of energies and angles at which the ions are directed towards the surface of the wafer, which is a result of the collisions in the background gas due to the applied process pressure [28,29]. The ion angular distribution function (IADF) is described as a Gaussian distribution. The angles, i.e., the IAD, under which the ions approach the substrate, are shown in Figure 5. As is explained by Sawin et al. [28,29], the IADF is dependent on the process pressure. Collision-induced scattering significantly increases at process pressures larger than 10 mTorr to 20 mTorr. Higher pressures, therefore, result in a change in the ratio of lateral etch to vertical etch, as the chemical flux is increased. The directionality of the etch process will also decrease due to the increased IAD. A larger IAD influences the area inside the scallop where the FC layer will be stripped prior to the etching of the next scallop. The effect of the pressure in the etch cycle step on the scallop dimensions is studied by varying the pressure in the etch step to 16 mTorr (being the lowest pressure achievable using all other settings), 30 mTorr, and 40 mTorr.

#### 2.2.2. Platen Power and Image Force

**Figure 9:**
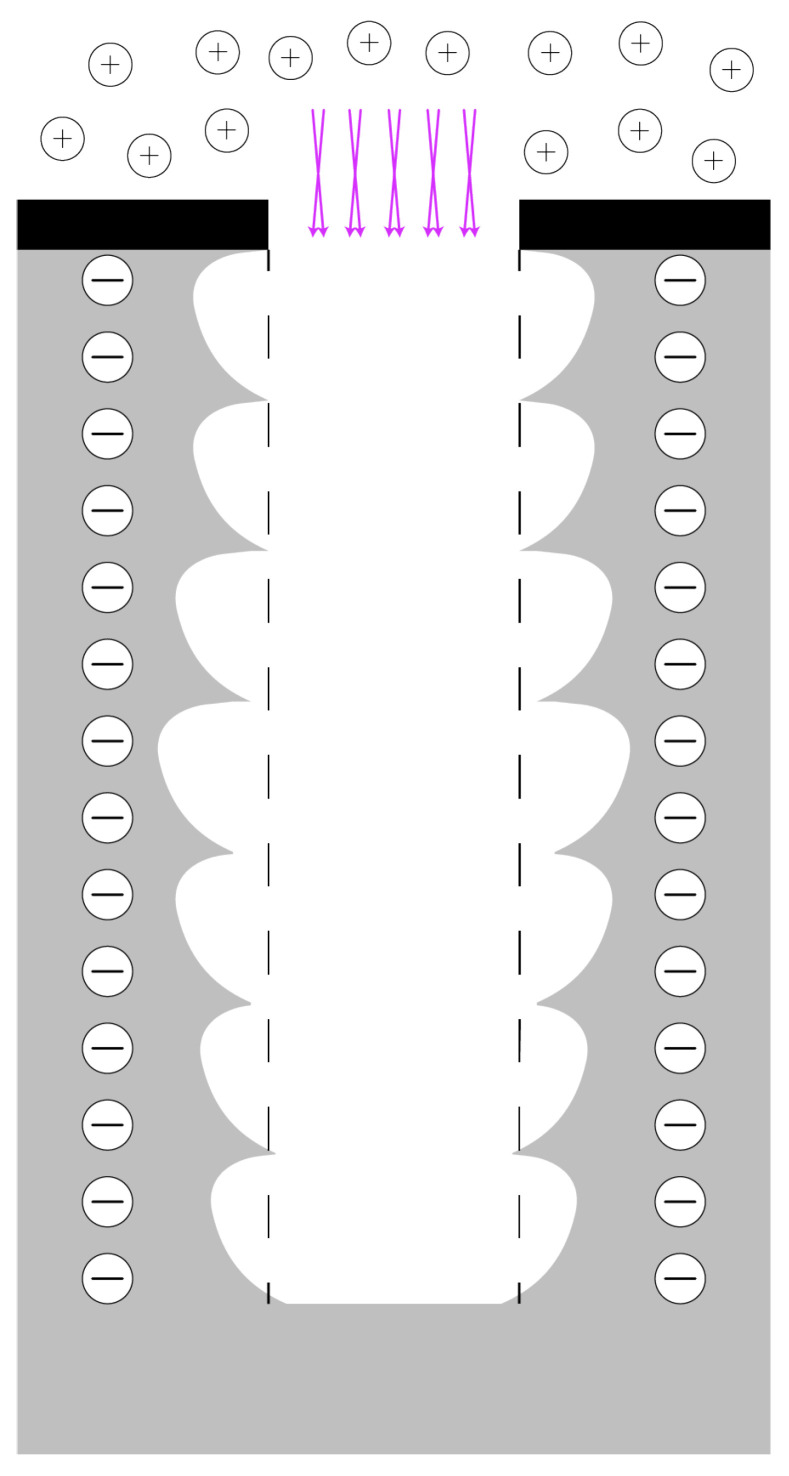
A schematic illustrating the changes in scallop dimensions and location inside the trench sidewall, thereby creating local widening in the middle of the trench (bottling effect) which is caused by a low platen power (CCP) setting and the IF. The IF electro-statically attracts the positively charged ions towards the sidewalls of the trench, changing the area at the bottom of the trench where FC is removed prior to the etch of the next scallop. The starting location of the next scallop moves more into the bulk, as is also depicted in Figure 5. This will result in a local widening of the trench ad/or a negatively tapered trench profile.

The power of the CCP and image force (IF) also affect the scallop’s dimensions. A process performed with too-low CCP power often results in a negative taper and/or local widening (also referred to as bottling) in trench structures (Figure 9) due to the so-called IF [33,34,35,36,37,38,39,40]. The IF is the attraction created by the negative potential of the conducting sidewalls with respect to the plasma. The IF attracting the positively charged ions scales inversely proportionally to the square of the distance between the ion and this sidewall. Therefore, ions moving relatively close to the sidewall are attracted with a greater force, which will deflect the ions towards the sidewall. This will increase the area at the bottom of the trench where the FC layer will be removed due to the inelastic collisions by the positively charged ions. Therefore, the starting point of the scallop is translated into the bulk of the silicon. As shown in Figure 9, this effect will result in a local widening somewhere along the length of the trench when the ion energy is not uniform along the trench depth (i.e., creating IF hotspots along the trench sidewall). Increasing the CCP increases the energy of the field, which, in combination with a low process pressure, results in a more biased trajectory of the ions towards the substrate and makes them less influenced by the IF. Therefore, the etch becomes more directional again. In this work, the low-frequency CCP (${\mathrm{CCP}}_{\mathrm{LF}}$) was varied between 22 W and 40 W.

#### 2.2.3. Trench Profile, Etch Cycle Time, and Ion Angular Distribution

In Figure 5, a model is presented that illustrates how the scallop size and location in the sidewall determine the position of the next scallop in the trench, thereby defining the profile angle of the trench sidewall. The gradient in the plasma, depicted as a purple gradient, in the pictures of the second row, indicates the Gaussian distribution of the IADF. For a small IAD, more ions are concentrated towards a directional beam, as indicated by the darker colour.

From the schematics in Figure 5a,b it is clear that by decreasing the IAD that one could obtain a directional etch. As illustrated in Figure 5c, the etch-cycle time is responsible for the length and width of the scallop. We demonstrated that by reducing the etch-cycle time; the scallop dimensions reduced and a positive taper was obtained, which are required for complete trench refilling by LPCVD. In this work, etch-cycle times of 1.50, 1.75, 2.00, 2.50, 3.00, and 5.00 s were used. The IAD and ion energy were further tailored to counteract image force effects and black silicon formation, by adjusting the process pressure and ${\mathrm{CCP}}_{\mathrm{LF}}$ during the etch step.

## 3. Materials and Methods

A 1 μm t-SiO_2_ hard mask layer for DRIE was grown on p-type (boron-doped with a resistivity of 5 Ωcm to 10 Ωcm), 100 mm, one-side polished (OSP) <100> silicon wafers of 525 μm thickness using a TS6304 furnace of Amtech Tempress Systems. This hard mask layer was patterned with 3 μm wide trenches (total loading of 1.45%) via conventional broadband ultraviolet photolithography in an EVG620 mask aligner using a 1.7 μm thick Olin OiR 907-17 photo-resist. The photo-mask was coated with a 50 nm DuPont^TM^ Teflon${}^{®}$ anti-stiction layer to enable the combined use of vacuum contact and hard contact to achieve a photo-resist profile angle of 88° to 90° without damaging the photo-resist layer upon contact. After exposure, the resist was developed in OPD-4262 developer, and the hard mask was etched by a directional CHF_3_/O_2_ RIE in a Plasma-Therm 790 to create a (near-) 90° sidewall angle in the t-SiO_2_ hard mask, which is of importance for high-quality HAR etching. The pattern was directionally over-etched to ensure full opening of all trench patterns (see Figure 7). The photo-resist was stripped using 99% HNO_3_, after which the HAR trenches are etched in an Oxford Instruments PlasmaPro100 Estrelas high-density dual-source ICP/CCP plasma etcher equipped with fast-switching Sensirion SFC4100 MFCs [41], using a standard two-step Bosch-based DRIE recipe for HAR MEMS structures, which etches silicon with a rate in the order of 1 μm/min^−1^ to 2 μm/min^−1^. After the HAR trench etch, an in situ FC strip was performed [42]. Remaining FC could lead to decreased layer adhesion between the silicon and refill material, and thereby could influence the mechanical strength of the device [43,44,45].

Within the used Bosch-based process, the chuck temperature was maintained at 25 ${}^{\circ }C$, helium backside cooling pressure was maintained at 10 Torr, and gas flows remained fixed (etch step: SF_6_/C_4_F_8_ 200 SCCM/10 SCCM; deposition step: SF_6_/C_4_F_8_ 10 SCCM/ 200 SCCM). A strike-up step was required to get a uniform start of the first entrance etch. The ${\mathrm{CCP}}_{\mathrm{LF}}$ was boosted for 30% of the etch-cycle time to ensure FC removal. The starting recipe is shown in Table 1, and the HAR trenches obtained using this recipe are shown in Figure 2 and Figure 4.

Inspection of the scallops was performed by breaking the wafer and inspecting the cross-sections using a JEOL JSM-7610F Schottky Field Emission SEM (JEOL Ltd., Tokyo, Japan). During the inspection, the trenches were inspected for trench depth and width, sidewall angle, and scallop width and height for the first ten scallops at the top of the trench (see Figure 6).

Furthermore, the effects of the scallop dimensions and sidewall profile angle on the refill quality were demonstrated by depositing a multi-layer stack consisting of 400 nm t-SiO_2_ grown using wet thermal oxidation at 1150 ${}^{\circ }C$ in a TS6304 furnace and a 2 μm thick low-stress poly-Si layer deposited by LPCVD at 595 ${}^{\circ }C$ in an Omega Junior furnace, both of Amtech Tempress Systems. The t-SiO_2_ resulted in good contrasts during SEM inspection afterwards. The temperature of 1150 ${}^{\circ }C$ was used to prevent sharpening of the corners (e.g., trench entrance and scallop tips) [46].

## 4. Results

### 4.1. Trench Entrance Etch

**Figure 10:**
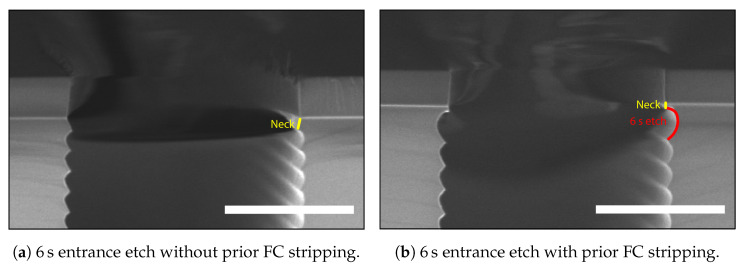
SEM images of the trench entrance etched with a 6 s isotropic entrance etch step. (**a**) No stripping of FC was performed after the RIE of the t-SiO_2_ hard mask. It was expected that the remaining FC from this recipe would prevent the widening of the entrance. (**b**) Stripping of FC was performed prior to the HAR etch. Here, a widened first scallop is visible. However, the neck above this scallop is smaller but still present. The scale bars in (**a**,**b**) are 1 μm.

**Figure 11:**
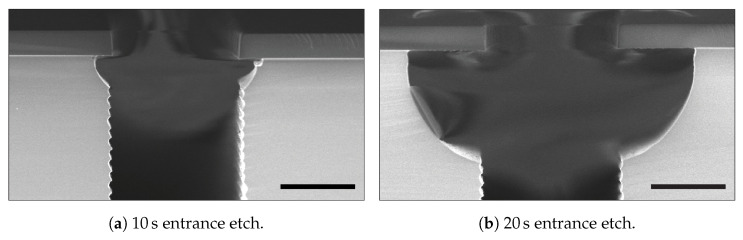
SEM images of the trench entrance etched with (**a**) 10 s of entrance etching and (**b**) 20 s of entrance etching. For both samples, the FC from the hard mask RIE was stripped prior to the trench etching. The neck was completely removed. However, rather large cavities were created. The scale bars in (**a**,**b**) are 2 μm.

As mentioned above, the width of the entrance has to be wider than the width of the trench below with positively tapered sidewalls. The entrance should not extend more than 1 μm on both sides of the trench, and the depth should not be deeper than 1 μm as well. The first, most straightforward approach to eliminate the neck in Figure 7 is by replacing the pre-deposition step of Table 1 with an isotropic entrance etch step. The result is shown in Figure 10 and Figure 11, achieved using trench entrance etch steps of 6, 20, and 20 s.

In Figure 10a, there is still a neck visible after a 6 s isotropic trench entrance etch. It is thought that the FC originating from the CHF_3_/O_2_-based RIE of the t-SiO_2_ hard mask prevented the widening of the entrance, thereby preserving the neck structure. The photo-resist strip had to be optimised to include stripping of FC. After the RIE of the hard mask, the wafers were cleaned using 30 min of oxygen plasma in a TePla 300 plasma processor, 2× 5 min 99 HNO_3_, quick dump rinsing in DI water, spin-drying, and a 30 s vapour hydrogen fluoride exposure to remove the formed SiO_*x*_ due to the oxidising behaviour of the oxygen plasma [47]. Figure 10b shows an SEM image after a 6 s trench entrance etch on a cleaned wafer. The neck was minimised but still present.

Longer times for the trench entrance etch resulted in the elimination of the neck, as shown in Figure 11. In Figure 11a, it is visible that it is hard to control the semi-isotropic shape just below the hard mask, as it is not symmetrical on both sides of the trench. This could result in difficulties during trench refill. The asymmetrical shape was removed (Figure 11b) by using a relatively long isotropic etch step. Using such entrances for a keyhole-free refill is possible. However, this creates rather extensive grooving on the wafers’ surfaces when they are refilled, as is shown in Figure 8a.

To overcome these limitations, a multi-step trench entrance etch was developed to eliminate the neck of Figure 7 and to control the length and width of the first two semi-isotropic shapes. This multi-step approach consists of two etch steps: a deposition step and two additional etching steps. The first etch step is performed at 40 mTorr and was used to clean up the area to be etched to avoid delays in the subsequent high-pressure etch and increase etch uniformity. Then, the second etch is performed at 100 mTorr. At this pressure, the chemical flux is dominant and a controlled semi-isotropic profile is etched. The deposition step is used to create a mask layer to protect the first isotropic shape and prevents extra widening. Two additional etch steps at 40 mTorr and 100 mTorr are performed to create a second, but smaller, semi-isotropic shape, which results in a v-shaped entrance profile. The shapes of these two semi-isotropic profiles can be adjusted by tuning the etch times of the four etch steps.

**Figure 12:**
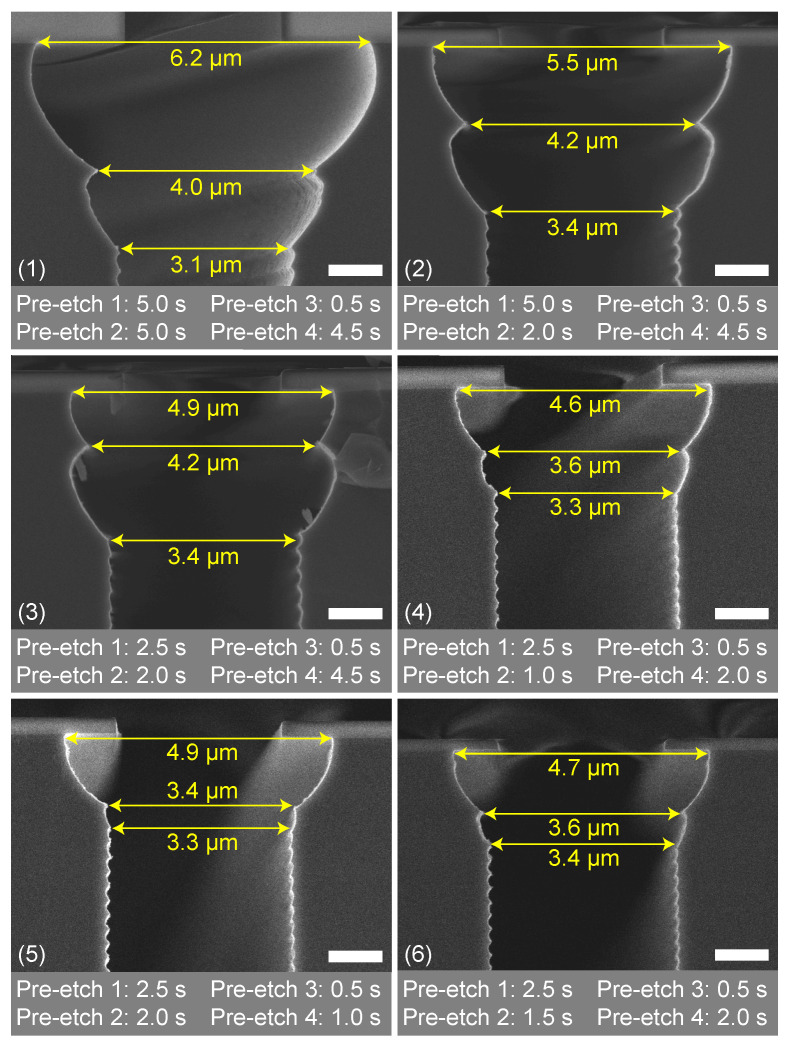
Optimisation of the trench entrance etch sequence. The deposition step between etch 2 and etch 3 was kept constant ( 2.4s). The numbers correspond to the experiment numbers in Table 2. All scale bars are 1 μm.

Figure 12 shows SEM images of the optimisation of the trench entrance etch for the used mask design. Each SEM image is denoted with the times of the four etch steps in the entrance etch sequence. The initial times of 10 s for step 1 and 2 combined and 5 s for step 3 and 4 combined (experiment 1 in Table 2) were chosen based on the results in Figure 11a. From that point on, times were shortened until the optimal entrance shape was obtained. Table 2 gives an overview of the used times. All other parameters in the etch steps were the same as in etch cycle of the Bosch-based recipe in Table 1. In Figure 12, it is demonstrated that the sizes of the two first scallops can be controlled by changing the times of the entrance etch steps, and therefore, the width of the under-etch beneath the mask. Such optimisation is dependent on the mask pattern (i.e., dependent on micro-loading [48,49,50,51,52,53,54,55,56] and macro-loading [38,40,49,55,57]), meaning that it has to be done only once per mask design.

As shown in Figure 8, the required entrance should have a positive taper, and the maximum depth and width should not be too significant, as this would result in undesired grooving at the surface. Based on the results in Figure 12, it can be concluded that sequences 4, 5, and 6 have similar results. However, trench entrance etch sequence 6, consisting of four etch steps (with in between the second and third etch step a deposition step) having times of 0.5, 1.0, 1.5, and 2.0 s were used for an optimal conformal refilling of 3 μm wide trenches, as this one had a slightly wider transition from the entrance region to the trench region.

### 4.2. HAR Trench Etch

To demonstrate the applicability of this Bosch-based etching of positively tapered trenches for a conformal refill, 3 μm trenches were etched 15 μm to 20 μm deep with a sidewall profile angle of 88° to 89°. The entrance and the HAR trenches were etched as a one-run process. After the fourth etch step of the entrance etch, the Bosch-based HAR etch continued and started with the etch cycle (see Table 3). It was expected that this would create a smooth transition from the entrance region to the trench region, as no further FC was deposited. The deposition step settings of the Bosch cycle were kept the same throughout all experiments. The cycle time, process pressure, and platen power during the etch cycle were used to examine the scallop width and length and their influences on the trench profile tapering. A complete overview of the recipe and the varied settings are given in Table 3 and Table 4.

#### Scallop Size and Etch Cycle Time

**Figure 13:**
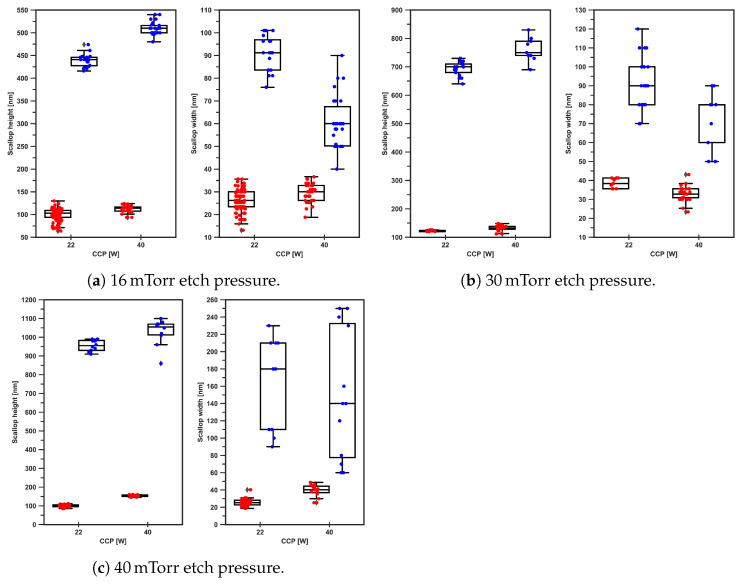
Graphs displaying the scallop height and width in nanometres as functions of etch-cycle time (1.5 and 5.0s), process pressures (16, 30, and 40 mTorr), and ${\mathrm{CCP}}_{\mathrm{LF}}$ settings (22 and 40 W). The colours correspond to: ● =1.5s and ● =5.0s.

The graphs in Figure 13 show the results as a relation between scallop width and height for 22 and 40 W${\mathrm{CCP}}_{\mathrm{LF}}$ platen power, 1.5 and 5.0 s etch-cycle times, and 16, 16, and 40 mTorr. Based on these graphs, the following relations can be found between the scallop height and width as a function of the parameter settings:

- **16 mTorr, 22 and 40 W for 1.5 and 5.0 s etch cycles:**
  For 1.5 s, the scallop height was in the order of 100 nm and for 5.0 s it was 450 nm to 500 nm. The ${\mathrm{CCP}}_{\mathrm{LF}}$ settings 22 and 40 W did not show a big difference for the height. For 1.5 s, the scallop width was in the order of 25 nm to 30 nm, and for 5.0 s, it is 60 nm to 100 nm.
- **30 mTorr, 22 and 40 W for 1.5 and 5.0 s etch cycle:**
  The same trend was observed for the scallop height and width when the pressure was increased up to 30 mTorr. The length of the scallops after 1.5 s etch steps was in the order of 125 nm, and for etch steps of 5.0 s this was 750 nm. The scallop height and width were not strongly affected by changing the ${\mathrm{CCP}}_{\mathrm{LF}}$ settings. This indicates that the higher pressure of 30 mTorr (or increased chemical flux) was responsible for the higher observed etch rate of silicon and the dimensional increase in the scallop, whereby the FC removal was not limiting this chemical flux.
- **40 mTorr, 22 and 40 W for 1.5 and 5.0 s etch cycle:**
  Increasing the pressure even further up to 40 mTorr increased the scallop height of the scallops for etch cycles of 5.0 s up to 950 nm. The width was less affected by the higher pressure and was for etch cycles of 5.0 s in the order of 60 nm to 240 nm. For 1.5 s, the influence was relatively small and resulted in dimensions comparable with those obtained at pressure values of 16 mTorr and 30 mTorr.

The strong increase in the scallop height for the 5.0 s etch-cycle time can be explained by a higher flux of chemical (fluorine) species. The physical (ion) flux, regulated by the ${\mathrm{CCP}}_{\mathrm{LF}}$ power and IAD, hasdno large effect on the scallop height for both 1.5 s and 5.0 s etch-cycle times. This indicates that the ${\mathrm{CCP}}_{\mathrm{LF}}$ power in this two-step Bosch-based process is high enough to remove the FC at the bottom of the trench and enable the subsequent etch of the scallop and that the ICP power and/or SF_6_ flow are not limiting the supply of chemical species.

The 1.5 s etch-cycle time controlled the scallop dimensions, as no big differences were observed between the different pressure values and ${\mathrm{CCP}}_{\mathrm{LF}}$ settings.

The large differences in the length and width of the etched scallops can be explained by the presence of the physical contribution (${\mathrm{CCP}}_{\mathrm{LF}}$ operated at 22 or 40 W) of ions at the bottom of the trench during the etch cycle steps, which enhanced the direction of the chemical reaction with silicon. For higher pressures, the impact of the ion flux with average lower ion energy was still high enough to remove the FC and etch the silicon.

**Figure 14:**
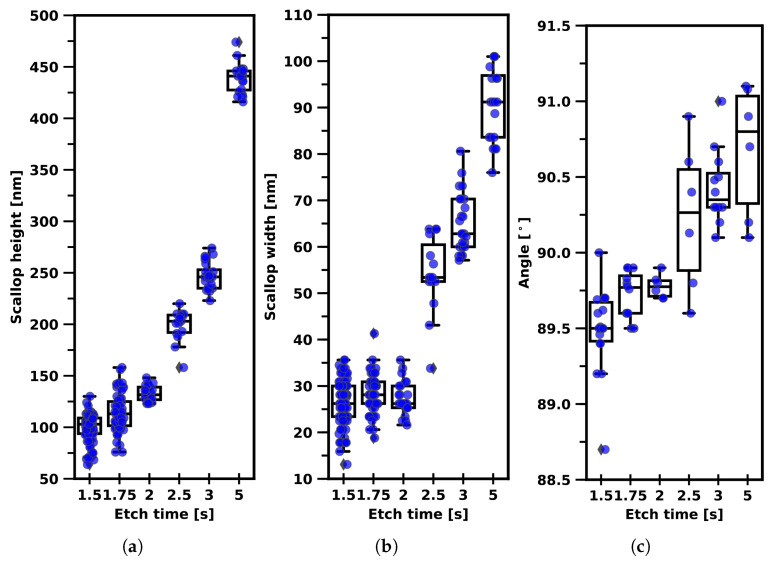
Graphs displaying the scallop height, scallop width, and trench sidewall profile angle for trenches etched with 22 W ${\mathrm{CCP}}_{\mathrm{LF}}$ platen power and 16 mTorr etch process pressure for different etch-cycle times. (**a**) Scallop height vs. etch time. (**b**) Scallop width vs. etch time. (**c**) Sidewall profile angle vs. etch time.

To demonstrate that the scallop dimensions are mainly controlled by the etch-cycle time, the etch-cycle time was varied from 1.5 s to 5.0 s, as is shown in Figure 14. This experiment is only useful when the FC removal is effective and the supply of chemical species (etch rate) is not limited by the SF_6_ gas flow and/or the ICP power [58]. The scallop height shows an exponential type of growth behaviour with increasing etch-cycle times due to the exothermic etch reaction of fluorine radicals with silicon. The scallop width and trench sidewall angle remained stable until the etch-cycle time exceeded 2.5 s. Below 2.5 s etch-cycle time, the trench sidewalls had the desired positive tapering of 88.0° to 89.5°. The scallop height was in the order of 100 nm, and the width was in the order of 30 nm.

**Figure 15:**
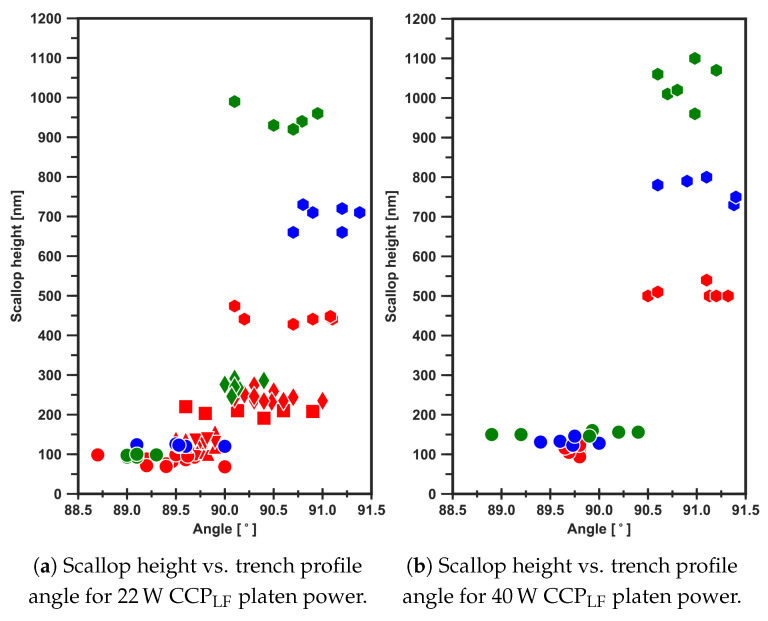
Two graphs with the relation of scallop height vs. trench sidewall angle for trenches etched with (**a**) 22 W and (**b**) 40 W ${\mathrm{CCP}}_{\mathrm{LF}}$ platen power for different etch process pressures and etch-cycle times. Legend: The figures are representing ● =1.5s, ▲ =1.75s, ▼ =2.0s, ■ =3.0s, and ⬢ =5.0s. The colours are representing ● =16mTorr, ● =30mTorr, and ● =40mTorr.

From the graphs in Figure 15, it is evident that the process pressure and platen power do not control the scallop dimensions of this recipe. For a long ( 5.0 s) etch-cycle time, the trench sidewall profile showed a negative tapering (re-entrant) and the angle was not influenced by these two settings; only the scallop height was. However, for shorter times, for example, 3.0 s at 22 W ${\mathrm{CCP}}_{\mathrm{LF}}$ platen power, the pressure did not influence the scallop height. In Figure 15, it is also shown that for etch-cycle times smaller than 2.5 s, the trench sidewall profile becomes positively tapered and is not influenced by the process pressure in this recipe for 3 μm wide trenches. This is in agreement with the results shown in Figure 14.

### 4.3. Trench Refill

**Figure 16:**
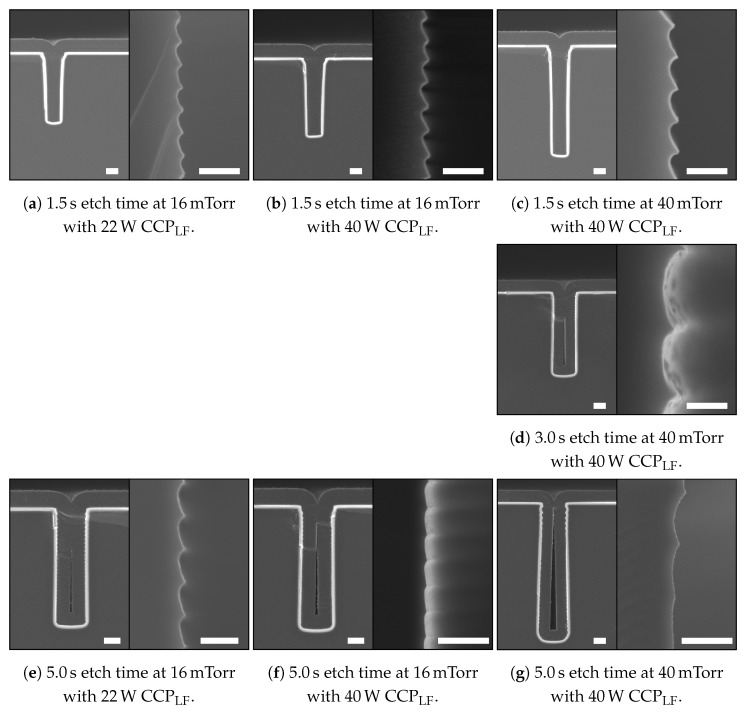
An overview of SEM images showing the relation of scallop dimensions on the refill of the trench. Each picture shows on the left side the complete trench, etched with different etch cycle settings and refilled with the multi-layer stack of 400 nm t-SiO_2_ and 2 μm poly-Si, and on the right side a close-up image of the scallops prior to the refill. These scallops were also used to construct the graphs of Figure 13, Figure 14 and Figure 15. The scale bars in (**a**–**d**) are 2 μm and 200 nm for the left and right figures, respectively. The scale bars in (**e**) are 2 μm and 500 nm for the left and right figures, respectively. The scale bars in (**f**,**g**) are 2 and 1 μm for the left and right figures, respectively.

Trenches 3 μm wide were etched 15 μm to 20 μm deep into a <100> silicon substrate using the listed etch settings to create different scallop heights and sidewall profile angles. To demonstrate the effects of scallop height and sidewall profile angle on trench refill, these trenches were refilled with a multi-layer stack as described in Section 3. The SEM images are shown in Figure 16.

Based on the SEM images in Figure 16, it can be observed that an etch-cycle time of 1.5 s results in relatively small scallops for the used mask design (i.e., loading). This etch-cycle time in combination with a limited chemical flux and small IAD will tune the trench sidewall profile towards being directional and positively tapered, as is shown in Figure 5c as well. The influence of the IF is suppressed by the low process pressure and an electric field induced by the ${\mathrm{CCP}}_{\mathrm{LF}}$, which gives the ions sufficient energy to overcome the attraction of the IF and ensure FC removal at the bottom of the trench.

Increasing the etch-cycle time and etch pressure will increase the contribution of the chemical flux. In this case, both the ICP power and SF_6_ flow during the etch step should be sufficient for the mask design/loading. This results in larger scallops and the sidewall profile tuning towards a negative tapering, as the relatively long etch-cycle time, in combination with the broad IAD, enables that the FC deeper in the scallops is also removed prior to the etch step. This localises the starting point of the next scallop deeper into the previous scallop and results in a negative tapering, as is illustrated in Figure 5a as well.

**Figure 17:**
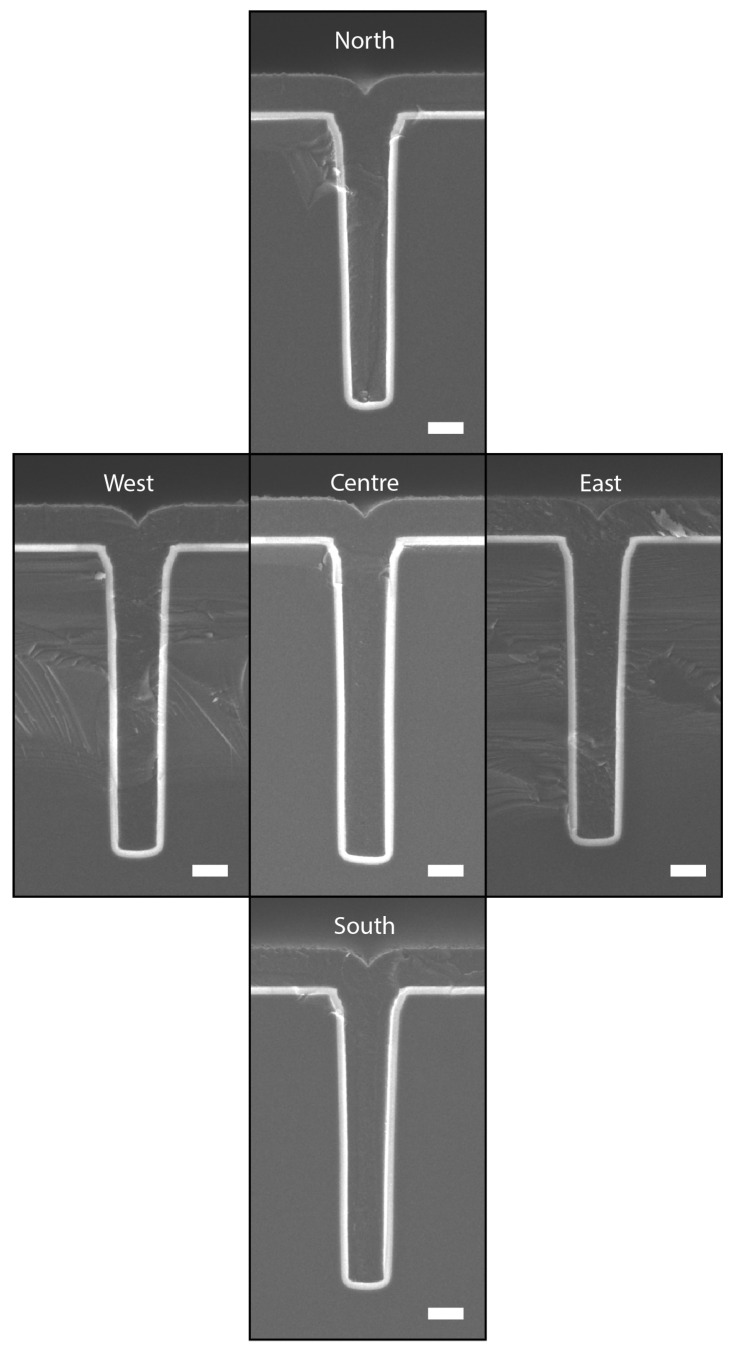
Cross-sectional SEM inspection of the wafer-scale uniformity in refilled trenches. Etch settings used during the trench etch were 1.5 s etch time at 40 mTorr process pressure and using 40 W ${\mathrm{CCP}}_{\mathrm{LF}}$ power. An aspect ratio of 6 was reached. The north, east, south, and west position were imaged at locations next to the 1 cm edge exclusion. All scale bars are 2 μm.

To demonstrate the wafer-scale uniformity of this method, a 5-point (centre, north, east, south, and west) cross-sectional SEM inspection with a 1 cm edge exclusion was performed on the wafer etched with the settings in Table 3, with etch-cycle time (**A**) being 1.5 s, etch platen power (**B**) being 40 W, and process pressure (**C**) being 40 mTorr. The results of this inspection are shown in Figure 17.

## 5. Discussion

In this work, the formation of the etched scallops in the sidewall of the trench was studied. These scallops were created by using a standard two-step Bosch-based recipe and can be used to tune the trench sidewall profile angle of HAR structures with dimensions of 3 μm in width and 15 μm to 20 μm in depth to the required angle of 88° to 89°.

**Figure 18:**
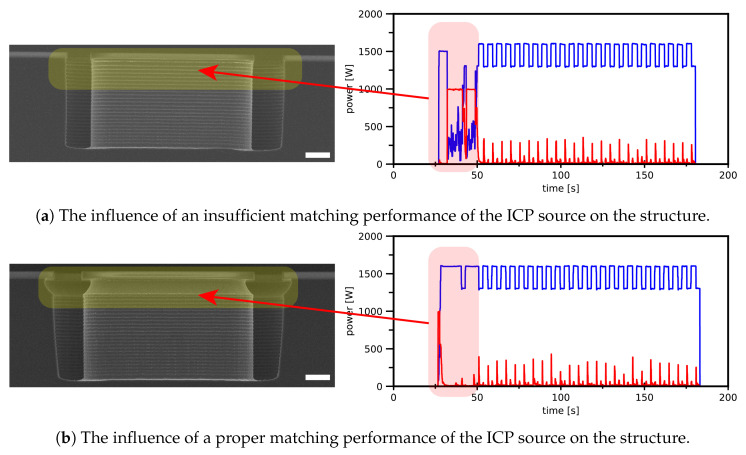
SEM images of test structures with their corresponding graphs displaying the inserted ICP power and power reflected during the process. Changing the pre-deposition step of the standard two-step Bosch-based recipe (Table 1) into the entrance etch sequence (Table 3) resulted in an unmatched ICP network, which influenced the results of the entrance etch to a great extent. Both scale bars are 3 μm. Colours represent: ▬ IPC power and ▬ reflected power.

The approach followed resulted initially in non-reproducible results during the entrance etch. The problem could be identified upon inspection of the log-files of the used DRIE system. The automatic matching unit of the ICP source had difficulties matching the impedance of the SF_6_ plasma, resulting in high reflected powers. The effect of efficient matching during the strike-up and the transition to subsequent steps on the resulting etch are shown in Figure 18. Therefore, it is advised to keep this in mind during process optimisation.

The used standard two-step Bosch-based recipe can be used to tailor the sidewall profile angle. However, the two-step architecture complicates the adjusting of the recipe, as in such recipes the FC removal and the semi-isotropic scallop etch are performed in the same process step, i.e., the etch cycle. To get better control of the shape and dimensions of the scallop, these two steps should be separated and have their own process settings [26,27]. FC removal requires different settings than the scallop formation. Preferably, it is done at low pressures in order to lower the IAD and at higher ${\mathrm{CCP}}_{\mathrm{LF}}$ powers to increase the physical ion flux, and therefore, increase the directionality. The most suited Bosch-based process to do this is the so-called DREM process [26,27]. In DREM, the FC strip is realised by a separate process step in between the deposition cycle and etch cycle. This makes DREM an ideal method for keyhole-free HAR trench refill applications. However, not all DRIE systems are equipped with such recipe architecture, and transferring a standard two-step process to a three-step DREM process requires some optimisation.

Another improvement to the HAR DRIE recipe presented in this work can be the ramping of process parameters during etching, as is incorporated in etch equipment of Surface Technology Systems Ltd. [59]. Gradually changing parameters can adjust the chemical flux (i.e., etch time and etch pressure) during etching, and therefore adjust the scallops in a more controlled fashion. However, this depends on the software interface of the machine. The used PlasmaPro100 Estrelas system is not equipped with this possibility.

In the experiments, IF effects were observed at ${\mathrm{CCP}}_{\mathrm{LF}}$ powers of <20W in combination with etch pressures of 30 and 40 mTorr. These results are excluded from this study as they clearly resulted in keyhole formation. In the final trenches, which were refilled, no IF effects were encountered, which indicates that the ion energy created by the ${\mathrm{CCP}}_{\mathrm{LF}}$ was high enough.

A final remark can be made on the schematic model presented in Figure 5. In these schematics, two effects were not taken into account. Firstly, possible influences of the IF (Figure 9) were not implemented. Secondly, with increasing aspect ratios, the ratio of chemical flux to physical flux changes. This will reduce the scallop size consequently, if a fixed set of process settings is used for the complete HAR DRIE process. This aspect ratio-induced change in scallop dimensions is also not taken into account. Both effects could be overcome by ramping process parameters, after which a more controllable Bosch-based process will be obtained.

## 6. Conclusions

In this work, several aspects regarding an optimal keyhole-free HAR trench refill were analysed and discussed. First of all, an analysis of the trench entrance was given. Two contributions, being a directional over-etch during the hard mask patterning and the architecture of the used Bosch-based HAR trench etch process, were identified as the origin of a necked entrance region. Using the proposed multi-step approach consisting of two sets of two isotropic etch steps with a deposition step in between, a tapered entrance region is obtained, which eliminates keyhole formation due to this necked entrance.

In this work, it was demonstrated that the scallop dimensions can be used to control the sidewall profile angle. It was demonstrated that the scallop height is the dominant dimension in the relation to the sidewall profile angle. The key parameter in tailoring the scallop height, and therefore the sidewall profile angle, is the time of the etch step in the Bosch cycle. This process parameter is subsequently used to tailor the sidewall profile angle of the HAR trenches to achieve a complete refill with a multi-layer stack of t-SiO_2_ and poly-Si.

The proposed method is a bulk micro-machining process which has no need for additional pre-processing steps, such as deposition and patterning of sacrificial layers; and post-processing steps, such as sacrificial layer stripping. The proposed method only has to be followed once per mask design (i.e., loading), making the proposed approach of interest to both academia and industry.

## Figures and Tables

**Table 1:** Starting recipe of the Bosch-based HAR DRIE in the PlasmaPro100 Estrelas system. The ${\mathrm{CCP}}_{\mathrm{LF}}$ was boosted for 30% of the etch-cycle time to ensure FC removal.

				Bosch Cycle	
Setting	Unit	Strike Up	Pre-Deposition	Deposition	Etching
**Temperature**	[${}^{\circ }C$]	25	25	25	25
**He BSC pressure**	[Torr]	10	10	10	10
**Time**	[s]	5.0	5.0	2.4	3.0
**ICP**	[W]	1500	1500	1300	1600
** ${\mathrm{CCP}}_{\mathrm{HF}}$ **	[W]	50	0	0	0
** ${\mathrm{CCP}}_{\mathrm{LF}}$ **	[W]	0	0	0	22
**Pressure**	[mTorr]	20	40	25	40
**C_4_H_8_ flow**	[SCCM]	50	200	200	10
**SF_6_ flow**	[SCCM]	0	10	10	200
**O_2_ flow**	[SCCM]	50	0	0	0

**Table 2:** Etch processes performed for controlled widening of the trench entrance.

		Experiments					
	Process	1	2	3	4	5	6
	Pressure	Time	Time	Time	Time	Time	Time
Step	[Torr]	[s]	[s]	[s]	[s]	[s]	[s]
**Etch 1**	40	5.0	5.0	2.5	2.5	2.5	2.5
**Etch 2**	100	5.0	2.0	2.0	1.0	2.0	1.5
**Deposition**	25	2.4	2.4	2.4	2.4	2.4	2.4
**Etch 3**	40	0.5	0.5	0.5	0.5	0.5	0.5
**Etch 4**	100	4.5	4.5	4.5	2.0	1.0	2.0

**Table 3:** The Bosch-based HAR DRIE with an optimised trench entrance etch.

			Trench Entrance Etch Sequence					Bosch Cycle	
Setting	Unit	Strike Up	Etch 1	Etch 2	Deposition	Etch 3	Etch 4	Etching	Deposition
**Temperature**	[${}^{\circ }C$]	25	25	25	25	25	25	25	25
**He BSC pressure**	[Torr]	10	10	10	10	10	10	10	10
**Time**	[s]	5.0	2.5	1.5	2.4	0.5	2.0	**A**	2.4
**ICP**	[W]	1500	1600	1600	1300	1600	1600	1600	1300
** ${\mathrm{CCP}}_{\mathrm{HF}}$ **	[W]	50	0	0	0	0	0	0	0
** ${\mathrm{CCP}}_{\mathrm{LF}}$ **	[W]	0	22	22	0	22	22	**B**	0
**Process pressure**	[mTorr]	20	40	100	25	40	100	**C**	25
**C_4_H_8_ flow**	[SCCM]	50	10	10	200	10	10	10	200
**SF_6_ flow**	[SCCM]	0	200	200	10	200	200	200	10
**O_2_ flow**	[SCCM]	50	0	0	0	0	0	0	0

**Table 4:** Optimisation settings for the etch cycle in the Bosch-based HAR trench etch.

Etch Cycle Setting		Unit	Values
**A**	Time	[s]	1.50, 1.75, 2.00, 2.50, 3.0, 5.0
**B**	Platen power ${\mathrm{CCP}}_{\mathrm{LF}}$	[W]	22, 40
**C**	Process pressure	[mTorr]	16, 30, 40

## Data Availability

Not applicable.

## Abbreviations

The following abbreviations are used in this manuscript:

C_4_F_8_	octafluorocyclobutane
${\mathrm{CCP}}_{\mathrm{HF}}$	high frequency capacitively coupled plasma
${\mathrm{CCP}}_{\mathrm{LF}}$	low frequency capacitively coupled plasma
CHF_3_	trifluoromethane
DI	de-ionised
DREM	Deposit, Remove, Etch, Multistep
DRIE	deep reactive ion etching
FC	fluorocarbon
HAR	high-aspect ratio
HNO_3_	nitric acid
IAD(F)	ion angular distribution (function)
ICP	inductively coupled plasma
IF	image force
LPCVD	low-pressure chemical vapour deposition
MEMS	micro-electromechanical systems
O_2_	oxygen
poly-Si	polycrystalline silicon
RIE	reactive ion etching
SEM	scanning electron microscopy
SF_5_^+^	sulfur pentafluoride cation
SF_6_	sulphur hexafluoride
Si_*x*_N_*y*_	silicon nitride (undefined)
SOI	silicon-on-insulator
(t-)SiO_2_	(thermal) silicon dioxide
TASCT	Trench-Assisted Surface Channel Technology
